# Recent advances in ferrocene-based nanomedicines for enhanced chemodynamic therapy

**DOI:** 10.7150/thno.101697

**Published:** 2025-01-01

**Authors:** Gui-long Wu, Senyou Tan, Xiaofeng Tan, Guodong Chen, Qinglai Yang

**Affiliations:** 1 Department of Hepatopancreatobiliary Surgery, The First Affiliated Hospital, Hengyang Medical School, University of South China, Hengyang, Hunan 421001, China.; 2 Center for Molecular Imaging Probe of Cancer Research Institute, Hengyang Medical School, University of South China, Hengyang, Hunan 421001, China.; 3 Department of general Surgery, Turpan City People's Hospital, Tulufan 838000, China.

**Keywords:** Ferrocene, Enhanced Chemodynamic therapy (ECDT), Synergistic therapy, Nanomedicine

## Abstract

Malignant tumors have been a serious threat to human health with their increasing incidence. Difficulties with conventional treatments are toxicity, drug resistance, and recurrence. For this reason, non-invasive treatment modalities such as photothermal therapy (PTT), photodynamic therapy (PDT), chemodynamic therapy (CDT), and others have received much attention. Among them, Ferrocene (Fc)-based nanomedicines for enhanced Chemodynamic Therapy (ECDT) is a new therapeutic strategy based on the Fenton reaction. Based on ferrocene's good biocompatibility, potentiation in medicinal chemistry, and good stability of divalent iron ions, scientists are increasingly using it as a Fenton's iron donor for tumor therapy. Such ferrocene-based ECDT nanoplatforms have shown remarkable promise for clinical applications and have significantly increased the efficacy of CDT treatment. Ferrocene-based nanomedicines exhibit exceptional consistency owing to their low toxicity, high stability, enhanced bioavailability, and a multitude of advantages over conventional approaches to cancer treatment. As a consequence, a number of tactics have been investigated in recent years to raise the effectiveness of ferrocene-based ECDT. In this review, we detail the different forms and strategies used to enhance Ferrocene-based ECDT efficiency.

## Introduction

The incidence of malignant tumors is surging, with the number of malignant tumor patients increasing to 28.4 million by 2040, posing a great danger to human health 1. The first-line cancer clinical treatments, including surgery, chemotherapy (CT), and radiotherapy, could partly cure the primary tumor while causing irreversible harm to the patient's body organs and tissues, leading to decreased immunity, severe pain, drug resistance, uncontrolled metastasis, and other adverse effects 2-9. Therefore, the rational design of non-invasive, efficient, precise, and biologically safer cancer treatment methods is of great significance for the future development of cancer therapy.

Recently, anticancer research has been gradually focusing on some emerging technologies, such as photothermal therapy (PTT) 10-15, photodynamic therapy (PDT) 16-20, gas therapy (GT) 21-27, and chemodynamic therapy (CDT) 28-34. For instance, Ding *et al.* have created novel compounds, such as PFW-DOX/glucose oxidase (GOD) 35 and SPN-TAPP-PCB4 36, which have expanded the possibilities for cancer detection and therapy. Among them, CDT is an innovative treatment strategy for sufficiently inhibiting tumor growth. CDT utilizes the Fenton/Fenton-like reaction that could *in situ* convert intratumoral hydrogen peroxide (H_2_O_2_) into highly toxic hydroxyl radicals (•OH) by transition metal ions in a weakly acidic environment (typical reaction scheme: Fe^2+^ + H_2_O_2_ → Fe^3+^ + •OH + OH^-^) 37-43. The •OH is the most reactive among the reactive oxygen species, which results in DNA damage and protein denaturation to cause senescence, death, and carcinogenesis of tumor cells 44-46. Notably, the Fenton/Fenton-like reaction would not work in normal tissues due to their neutral pH and insufficient H_2_O_2_ concentration, thus allowing for highly selective tumor therapy 47-49. Therefore, CDT has several manifest superiorities: low invasiveness, superior selectivity, and fewer adverse effects. Nevertheless, there are still some problems that hinder the further development of CDT. First, the Fenton/Fenton-like reaction should have exerted its highest stage in an acidic environment (pH at 2-4), while the pH of solid tumors is at approximately 6.5, constraining an efficient intratumoral Fenton/Fenton reaction 37, 50, 51. Second, the limited H_2_O_2_ level inside the cancerous tissue cannot afford to produce •OH continuously, thereby rendering individual CDT treatments ineffectual. Moreover, antioxidants within cells possess a powerful scavenging effect on short-lived, highly reactive •OH and diffusion distance, further limiting the antitumor efficiency. In addition, persistent harm to tumor cells in a short amount of time by •OH depends on its generation rate, which is mainly limited by the performance of the catalyst 52-54. Therefore, optimizing the effectiveness of CDT by designing innovative catalysts and promoting their composition and organization would be beneficial to potential clinical cancer therapy 55.

Metallocene is an organometallic complex by doping metal ions into organic scaffolds. The ferrocene as the typical of metallocene was discovered in 1951 and determined with a sandwich structure [Fe(η^5^-C_5_H_5_)_2_] by Wilkinson and Fischer owing to the formation of a symmetrical covalent bond with the d-orbitals of Fe^2+^ cation, which were awarded the Nobel Prize in chemistry in 1973 56, 57. The non-toxic and lipophilic nature of ferrocene makes it an attractive core for grafting with bioactive pharmacophores to influence their biological activity and confer drug-like properties. With the development of nanotechnology in medicinal chemistry, cancer nanomedicines based on nanomaterials and devices to realize drug delivery, diagnosis and imaging, and synthetic vaccine development open a new chapter for the human fight against cancer. Ferrocene-based nanomedicine has been extensively reported in the biomedical field owing to the nature of low toxicity, high stability, and reversible redox in non-oxidizing environments 58, 59. Ferrocene-based nanomedicine mainly triggers the generation of •OH to exert anticancer effects by the Fenton reaction between the ferrocene group and H_2_O_2_ in the tumor microenvironment. In turn, the process of reaction would result in the creation and build-up of cytotoxic quinone-methide, which causes DNA damage or apoptosis via an intracellular redox event 60. Furthermore, the moiety of ferrocene increases the lipid solubility of the overall nanomedicine and facilitates their delivery to the desired targets 55. However, single CDT therapy based on ferrocene-based nanomedicine has not been very effective for complete tumor elimination. Therefore, scientists are currently focusing on enhanced chemodynamic therapy (ECDT) based on ferrocene-based nanomedicine. The use of ferrocene in ECDT combination therapy presents both challenges and opportunities. The main challenges are that ferrocene as an organic small molecule is difficult to apply to biological systems, and the selective delivery of Fe^2+^ to tumor sites is also challenging, and this has become a challenge for the ECDT application of ferrocene molecules 61, 62. However, at the same time, the iron cyclopentadienyl (Fe-Cp) bond has a large dissociation energy (≈ 90 kcal mol^-1^), which is less prone to be oxidized to the trivalent state (Fe^3+^) than the ionic state of Fe^2+^ 63. This unique advantage makes ferrocene-based nanomedicines uniquely advantageous for ECDT biologic applications.

**Scheme 1:**
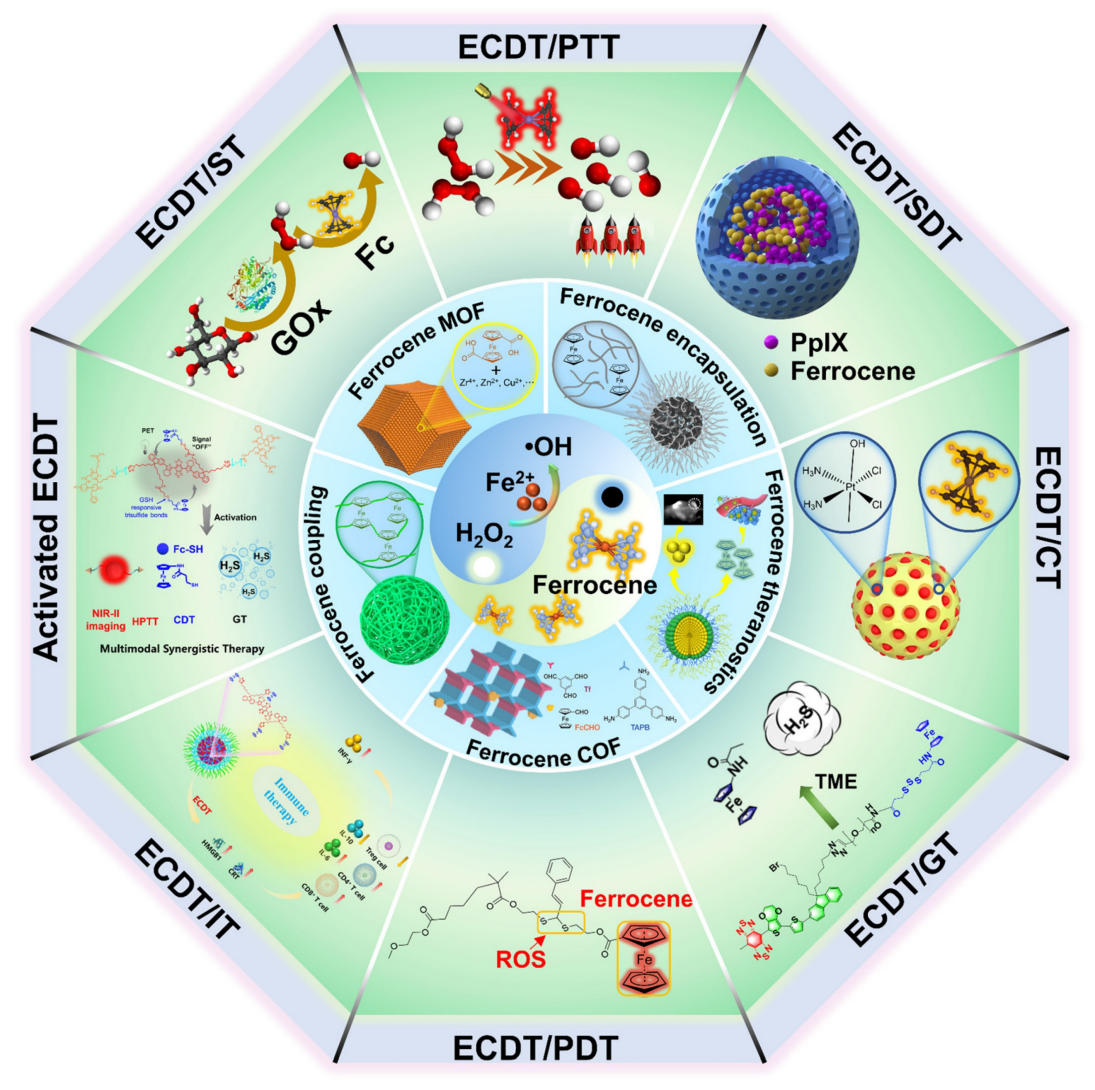
The schematic illustration for the type of Ferrocene-based nanomedicines and applications of enhanced CDT.

Considering the significance of ECDT anticancer tactics and the existing gaps in reviews in the field, there is a pressing requirement for a thorough analysis of the most recent developments in ferrocene-based nanomedicines for ECDT. In this work, we first summarize the recent significant progress in different ferrocene-based nanomedicines with different forms of ferrocene molecules, such as ferrocene-encapsulated nanoparticles, ferrocene-coupled nanoparticles, ferrocene-based nanotheranostics, ferrocene-based metal-organic frameworks (MOF), and ferrocene-based covalent organic frameworks (COF) (**Scheme 1**). Then, we comprehensively review the recent remarkable advances in ferrocene-based ECDT synergistically with other therapeutic models and explicitly describe the design advantages and possibilities for the clinical application of the state-of-the-art ferrocene-based nanoplatforms. Finally, we exhaustively analyze the existing challenges, constraints, and potential advancements of ferrocene-based nanomedicines for ECDT.

## 2. Different kinds of ferrocene-based nanomedicines for CDT

In recent years, inspired by the development in nanotechnology and in-depth comprehension of the ECDT mechanism 31, researchers have attempted to construct efficient and precise ferrocene-based nanomedicines in chemodynamic cancer therapy. However, to the best of our knowledge, there is no comprehensive review for exploring the structural forms of different ferrocene-based nanomedicines and their effects. Therefore, this section will specifically discuss the structure-activity relationship between the newly developed ferrocene-based nanomedicines and tumor therapy applications based on them. These types of ferrocene-based nanomedicines are summarized as follows: ferrocene-encapsulated nanomedicines, ferrocene-coupled nanomedicines, ferrocene-based nanotheranostics, ferrocene-based metal-organic frameworks (MOF), and ferrocene-based covalent organic frameworks (COF).

### 2.1 Ferrocene-encapsulated nanomedicines

**Figure 1:**
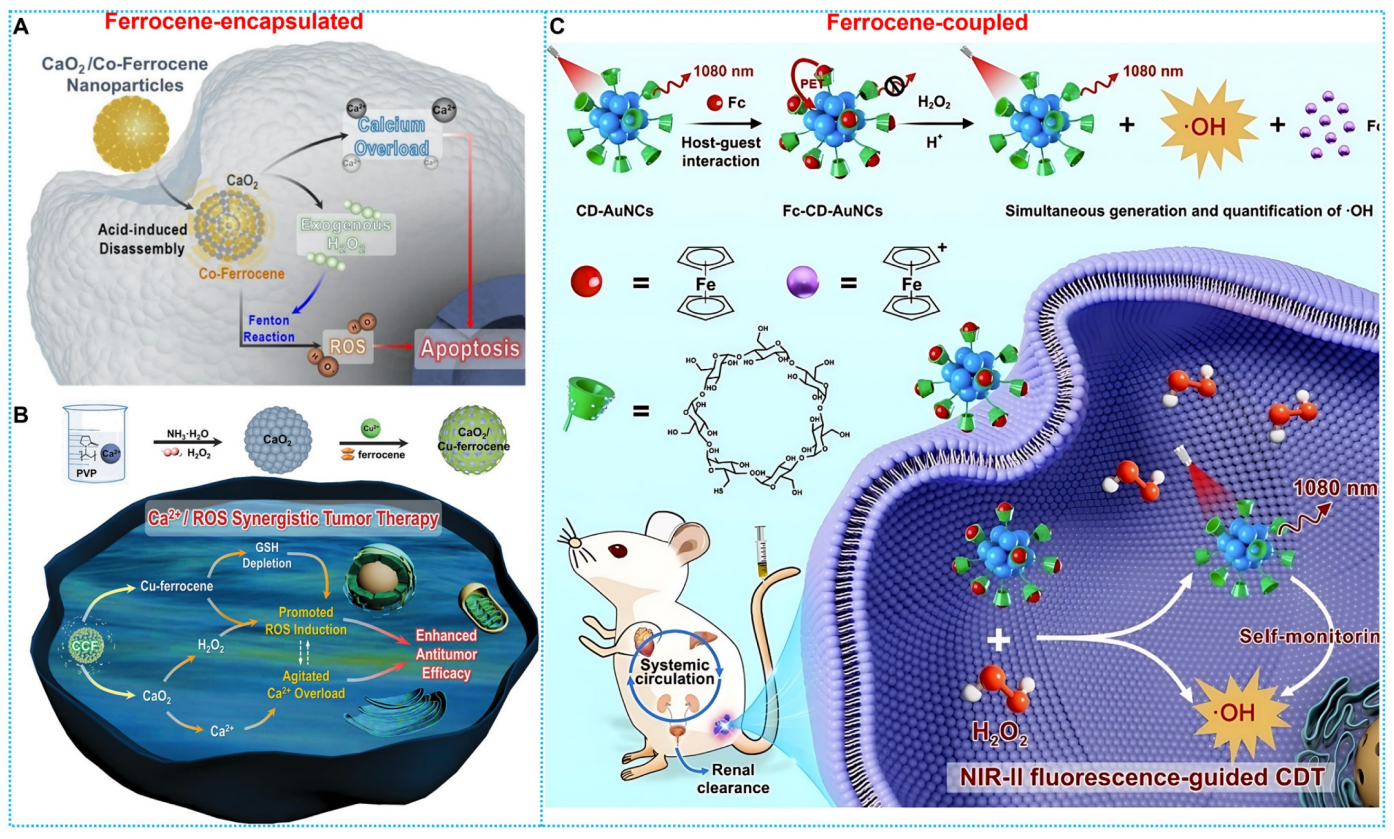
(A) Synthesis and therapeutic mechanisms of CaO_2_/Co-Ferrocene NPs. Reproduced with permission from ref 64. Copyright 2021, Springer. (B) Working principle and preparation of CaO_2_/Cu-ferrocene NPs. Reproduced with permission from ref 65. Copyright 2021, Wily. (C) Synthesis route and workflow of Fc-CD-AuNCs. Reproduced with permission from ref 66. Copyright 2024, Wily.

Based on the good biosafety and stability of ferrocene, researchers have developed ferrocene-based nanomedicines by encapsulating ferrocene in the form of loading or self-assembly. After these ferrocene-based nanomedicines enter the tumor cells, the ferrocene molecules would be released and react with H_2_O_2_ within the tumor to generate •OH to induce cell apoptosis. For instance, Zhou and colleagues developed a ferrocene-based nanoplatform CaO_2_@Co-Fc, which could decompose CaO_2_ and produce large quantities of H_2_O_2_. This strategy resulted in calcium overload and ferrocene-based CDT at the tumor site, and the combined effect resulted in a favorable tumor-suppressive effect (**Figure 1A**) 64. Another typical paradigm is reported by Kong and coworkers, who developed CaO_2_ nanoparticles that are loaded with Cu-ferrocene molecules at the surface (CaO_2_/Cu-ferrocene, CCF). The synergistic effect of the three metal ions, copper, iron, and calcium, produced a good tumor suppression effect (**Figure 1B**) 65. Furthermore, Lin and Chen's team 66 designed host-guest supramolecular Fc-CD-AuNCs for NIR-II fluorescence-guided cancer CDT by integrating the Fenton, redox, and PET activities of Fc (**Figure 1C**).

### 2.2 Ferrocene-coupled nanomedicines

The encapsulated-typed ferrocene usually confronts the leakage problem during the preparation, storage, and subsequent internal circulation process of nanomedicines, which aggravates the concern about biological safety and reduces the eventual treatment effect. Moreover, the Fenton or Fenton-like reaction could generate •OH by catalyzing H_2_O_2_ with highly dynamic low-valence Fenton metal ions while easily converting to high-valence (e.g., Fe^2+^ to Fe^3+^ and Cu^+^ to Cu^2+^) state with low activity after reaction 67. In this process, the leakage of metal ions becomes a key factor in affecting the efficiency of the Fenton reaction and clinical conversion 68. Chemically coupled ferrocene-based nanomedicines provide significant biosafety optimization as well as the potential to address molecular stability and leakage. Therefore, the chemical coupling strategy is employed to improve the efficiency of the Fenton reaction by increasing the single-molecule ferrocene loading and avoiding molecular leakage. In addition, although exhibiting excellent ferrous ion stability and low toxicity, ferrocene's poor biocompatibility and hydrophobicity could not afford its clinical conversion application. Consequently, Strategies for coupling ferrocene to amphiphilic bioactive can effectively enhance the ability to change the hydrophobic properties of ferrocene organic molecules and thus improve their applicability in biological environments.

For instance, Ding and colleagues synthesized two polymer-modified ferrocene-based nanomedicines named PFW-DOX/GOD (DOX: doxorubicin, GOD: glucose oxidase), which exhibited minimal adverse effects and high accumulation at the tumor site. The disruption of the pH-sensitive benzoicimine bond will cause the dismantling of PFW-DOX/GOD. The declared GOD catalyzes the breakdown of intratumoral glucose to produce a significant amount of gluconic acid and H_2_O_2_, which both deplete the tumor's energy source and generate hydroxyl radicals via the Fenton reaction 35. Another typical example is reported by Xu and coworkers, who developed a self-immolated amphiphilic poly(ferrocenes) named BP*^nbs^*-Fc, which is made up of self-immolated framework and aminoferrocene (AFc) side chains as well as end-capping poly (ethylene glycol) monomethyl ether. Following the tumor's acidic environment, the BP*^nbs^*-Fc nanoparticles break down easily, releasing components of azaquinone methide (AQM), which may effectively exhaust glutathione (GSH) to cascade discharge AFc. Thereby raising the risk of oxidative damage to the cancerous cells 69. In addition, the team led by Sun has created a DNA-adjuvant hydrogel-optimized enzymatic cascade that accurately regulates the distance between glucose oxidase and ferrocene, which has resulted in a substantial improvement in the production of reactive oxygen species (ROS), leading to more efficient destruction of tumor cells. This DNA-adjuvant hydrogel-optimized enzymatic cascade has the potential to effectively treat solid tumors by enhancing the combined effects of CDT and immunotherapy 70.

### 2.3 Ferrocene-based phototheranostic platform

**Figure 2:**
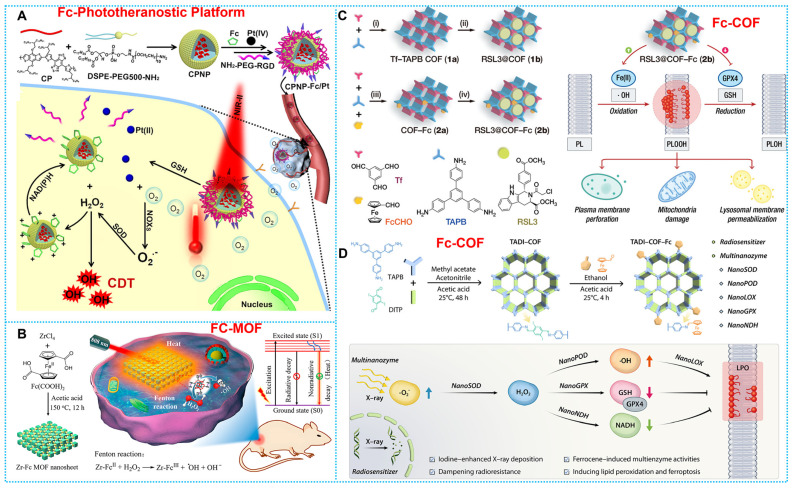
(A) Working principle and preparation of CPNPs. Reproduced with permission from ref 79. Copyright 2021, ACS. (B) Synthesis and therapeutic mechanisms of Zr-Fc MOF. Reproduced with permission from ref 92. Copyright 2020, ACS. (C) Synthesis route and workflow of RSL3@COF-Fc. Reproduced with permission from ref 93. Copyright 2021, Wily. (D) Schematic diagram of therapeutic mechanisms of TADI-COF-Fc. Reproduced with permission from ref 94. Copyright 2023, Wiley-VCH.

The ferrocene-based nanomedicines play an indispensable role in cancer therapy. Before initiating treatment of various cancers, it is essential to carry out diagnostic imaging to understand the cellular phenotype(s) and heterogeneity of the tumor. On this concept, the construction of ferrocene-based nanotheranostics is emerging as a revolutionary and promising method for cancer therapy. The clinical interventions used today are out of step with the clinical diagnosis, restricting tumor treatment's effectiveness and precision to some degree 71. The ultimate goal of ferrocene-based nanotheranostics is to gain the ability to image and monitor the cancer tissue, delivery kinetics, and treatment efficacy with the long-term hope of gaining the ability to tune the therapy. In light of its excellent specificity and sensitivity, fluorescence imaging (FLI) has generated a great deal of interest in the diagnostic field 72-74. The biological imaging window has steadily increased from the visible light area (400-700 nm) to the first area of near-infrared (NIR-I, 700-900 nm) and now to the second near-infrared region (NIR-II, 1000-1700 nm). Consequently, making use of integrated cancer theranostics is essential for combating cancer and raising the chances of recovery and survival for patients. Combined ferrocene-based therapeutic agents with NIR-II imaging connect therapeutic approaches with deep-tissue diagnostic imaging in a synergistic way that promises simultaneous precision therapy and early cancer detection 75, 76. Hence, Wei and coworkers established a rare-earth-nanocrystal (RENC)-based Fe^2+^ transfer device using light-control strategies and DNA nanotechnology to achieve controllable Fe^2+^ distribution. RENC's unique design provides the transport framework with the capacity to both regulate and diagnose deliveries 77. In addition, organic conjugated fluorescent molecules, with their excellent luminescence performance and carbon structure of good biocompatibility, are also excellent diagnoses and treatments. For example, Wu and coworkers developed IR-FEP-RGD-S-S-S-S-Fc, a NIR-II organic phototheranostic platform activated by the high GSH concentration Tumor microenvironment. The intrinsic fluorescence of IR-FEP-RGD-S-S-S-S-Fc is removed via photoinduced electron transfer (PET) between NIR-II fluorescence cores with ferrocene, and the fluorescence signal is triggered when the cleavable linker unit breaks in the presence of the high GSH concentration Tumor microenvironment. This "activatable" biotargeting NIR-II organic photodiagnostic platform reduces the unanticipated toxicity of the substance and provides a theoretical framework for the creation of highly accurate tumor-targeting combination therapy approaches 78. Furthermore, He *et al.* constructed a nano-amplifier CPNP-Fc/Pt by self-assembly of conjugated polymer nanoparticles (CPNPs) modified with ferrocene (Fc) and cisplatin prodrugs (Pt (IV)). The CPNPs showed a strong photothermal response when exposed to light at 1064 nm. Meanwhile, Fe (II) in Fc exhibits more readily reversible redox properties compared to free Fe^2+^; thus, intracellular nicotinamide adenine nucleotide phosphate (NADPH) can regenerate Fc by reducing the Fenton reaction product, Fe (III) to Fe (II). The effective restoration of Fc further enhanced the CDT (**Figure 2A**) 79.

### 2.4 Ferrocene-based metal-organic frameworks (MOFs)

The first MOFs were created with a kind of organic-inorganic porous material by the coordination between metal ions and organic ligands 80. The movable framework, high porosity, facile functionalization, and stimuli-responsiveness of metal-organic frameworks (MOFs) have expanded their potential biological applications 81, encompassing bioimaging 82, antitumor 83, anti-bacterial 84, and biocatalysis 85. Concretely, MOFs' variable selection towards coordinated metal ions and induced sensitivity to the TME make them ideal options for CDT agents. Meanwhile, MOFs' excess porosity and transparency allow them to be employed as transporters for a variety of species, from medicines to diagnostics, where the release of the functional molecules enhances the start and enhancement of CDT under the influence of the TME. Moreover, MOFs may be used as direct chemodynamic agents when their building blocks are active compounds with Fenton/Fenton-like reaction capabilities 86-88. MOFs were usually employed as carriers by employing their porosity for delivering and releasing a variety of functional molecules 89, 90. Ferrocene derivatives (such as 1,1'-Ferrocenedicarboxylic acid (Fc(COOH)_2_) as ligands can form multifunctional MOF nanomaterials by binding multiple metal ions, showing good potential for tumor therapy. For instance, Fang *et al.* constructed a Co-Fc NMOF incorporated with GOx molecules named Co-Fc@GOx. In this platform, along with the plentiful H_2_O_2_ production and the reduction in pH by GOx catalysis, the limiting parameters of the Fenton reaction were thoroughly optimized 91. Another typical paradigm is reported by Deng and coworkers, who developed a therapeutic platform that combines PTT and CDT in harmony via the Zr-Fc MOF nanosheet application. The Fc(COOH)_2_ ligand endows the Zr-Fc MOF nanosheet with catalytic activity to facilitate the production of •OH for CDT **(Figure 2B)** 92. The studies mentioned above indicated that the loose and porous structural units of MOFs possess beneficial compounds with Fenton/Fenton-like reactivity. This fundamentally enhances the efficiency of the Fenton reaction of ferrocene. Additionally, this unique property also allows for the possibility of modifying and loading multiple substances, thereby improving the therapeutic effectiveness of Ferrocene-based nanomedicines through multiple synergistic effects.

### 2.5 Ferrocene-based covalent organic frameworks (COFs)

Being different from metal-organic frameworks (MOFs), COFs belong to a new type of crystalline porous materials, which are composed of light elements and linked by robust covalent bonds. COFs have garnered a lot of interest in biological research because of their high surface area, changeable pore size, and low density 60. Based on this concept, COFs have been employed by Fenton/Fenton-like catalyst doping in Ferrocene-based nanomedicines. For instance, Zhou *et al.* developed a COF-based therapeutic system called RSL3@COF-Fc. Once RSL3@COF-Fc is endocytosed by tumor cells, the progressively released methyl (1*S*,3*R*)-2-(2-chloroacetyl)-1-(4-(methoxycarbonyl)phenyl)-2,3,4,9-tetrahydro-1*H*-pyrido[3,4-*b*]indole-3-carboxylate (RSL3) inhibits GPX4 (a central checkpoint of the antioxidant system of the tumor cells) to perturb the redox homeostasis, whereas the Fc induces the production of •OH via Fenton-like reactions, leading to lipid peroxidation (**Figure 2C**) 93. Another typical example is developed by Zhou *et al.*, who designed a multifunctional COF nanozyme (TADI-COF-Fc), which features iodine atoms and ferrocene (Fc)-based for use in treating radioresistant esophageal carcinoma in conjunction with radiation therapy. TADI-COF-Fc not only generates multiple reactive oxygen species (ROS) *in situ* but furthermore produces H_2_O_2_ from superoxide, which results in decreased nicotinamide adenine dinucleotide (NADH) dehydrogenation, lipid peroxidation, glutathione (GSH) oxidation, and the generation of •OH. Eventually, increment in cancer cell death (**Figure 2D**) 94. The above investigations have verified that crystalline porous structural units in COFs, similar to MOFs, exhibit enormous activity as compounds with Fenton/Fenton-like reactivity. COFs composed of light elements possess desirable characteristics such as high specific surface area, variable pore size, and low density. These properties enable multiple modification options and excellent biocompatibility for the Ferrocenyl Fenton reaction.

## 3. Ferrocene-based synergetic enhanced chemodynamic therapy

Based on the above design mechanisms and synthesis methods, different types of ferrocene-based ECDT nanoplatforms have been established and have become a hot area for tumor therapy combined with other efficient modalities and strategies, such as ECDT in combination with starvation therapy (ST), ECDT in combination with chemotherapy, ECDT in combination with PTT, ECDT in combination with PDT, ECDT in combination with tumor TEM-activated therapy, ECDT in combination with Sonodynamic Therapy (SDT) and gene therapy, ECDT in combination with immunotherapy (IT), and ECDT in combination with gas therapy (GT). In addition, Chen and Lin's team found that ECDT can be achieved through various strategies such as modulation of cell membrane unsaturation and down-regulation 95 of ferritin heavy chain 96. We present the latest Ferrocene-based nanomedicine types, ECDT mechanisms, and related enhancement strategies categorized in **Table 1**.

### 3.1 ECDT in combination with ST

Starvation therapy is mainly used to “starve” tumor cells to death by blocking the nutritional supply to the tumor. Most works have focused on harnessing the potential of GOx-based catalytic reactions for CDT co-enhancement strategies 112-116, which provides a non-invasive strategy for cancer therapy to cut off the energy source of the tumor 117, 118. Moreover, the generation of H_2_O_2_ via the glucose oxidase (GOx)-mediated catalytic pathway significantly amplifies tumor oxidative stress, thereby inducing the destruction of cancer cells 112, 119. In addition, the generation of gluconic acid would decrease the intratumor pH to amplify the CDT effects.

**Figure 3:**
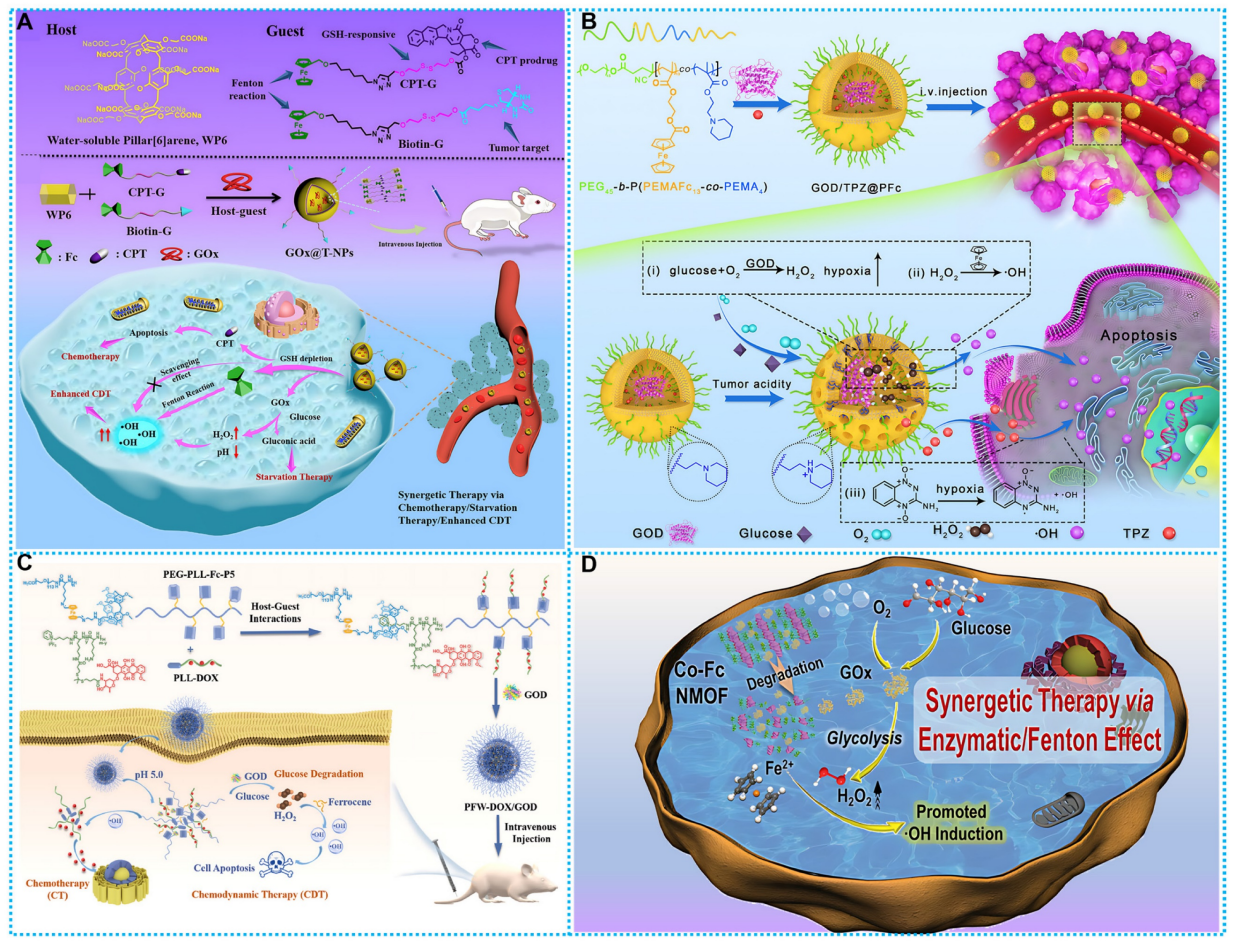
(A) Synthesis and therapeutic mechanisms of GOx@T-NPs. Reproduced with permission from ref 97. Copyright 2021, ACS. (B) Working principle and preparation of GOD/TPZ@PFc. Reproduced with permission from ref 98. Copyright 2021, Elsevier. (C) Synthesis route and workflow of PFW-DOX/GOD. Reproduced with permission from ref 35. Copyright 2022, Elsevier. (D) Schematic diagram of therapeutic mechanisms of Co-Fc NMOF. Reproduced with permission from ref 91. Copyright 2020, Wiley-VCH.

Drawing from these exhilarating advantages, Liu and coworkers successfully prepared a columnar 6-fluorene-based supramolecular GOx@T-NPs for rapidly facilitating the transformation of glucose to gluconic acid and H_2_O_2_. The multimodal synergistic effect of ferrocene-initiated CDT, starvation therapy ST (GOx), and chemotherapy (CPT) was achieved (**Figure 3A**) 97. However, this work could not avoid the problem that the hypoxic tumor microenvironment constrains the efficiency of H_2_O_2_ generation. Further, Wang *et al.* developed a synergistic pH-responsive polymerset nanoreactor of glucose oxidase (GOD) and tirapazamine (TPZ), GOD/TPZ@PFc, which not only generates the highly toxic •OH through ferrocene-based Fenton reaction but also produces •OH from the anticancer prodrug TPZ, under hypoxic conditions. The antitumor medication TPZ may be distributed and enabled to create more •OH when the hypoxic situation develops after O_2_ is depleted (**Figure 3B**) 98.

Similarly, Ding *et al.* developed a supramolecular peptide nano-drug (PFW-DOX/GOD). The coating of PEG sections and mean hydrodynamic diameter of 200 nm of PFW-DOX/GOD resulted in significant tumor retention with little adverse effect. In an internal acidic environment, PFW-DOX/GOD exhibits pH-triggered disintegration, distributing GOD, which then catalyzes intratumoral glucose into H_2_O_2_. After that, the Fenton reaction transforms sufficiently H_2_O_2_ into •OH, which is highly toxic to CDT. This strategy activated free doxorubicin (DOX) and glucose degradation to achieve synergistic antitumor effects of CDT and CT (**Figure 3C**) 35. Further, nanoscale ferrocene copolymer MOF was first discovered by Fang and coworkers. Co-Fc NMOF showed unique Fenton activity, which was mainly derived from Fe^2+^ containing ferrocene. The unique cascade enzyme/Fenton effect facilitated CDT and ST. Demonstrated superior ability in cancer cell inhibition and tumor regression (**Figure 3D**) 91.

### 3.2 ECDT in combination with CT

Chemotherapeutic agents with *in situ* self-supply of H_2_O_2_ are ideal supplements to replenish CDT. Some anticancer chemotherapeutic drugs, such as doxorubicin (DOX) and cisplatin, could produce •O_2_^2-^ and subsequently converted by superoxide dismutase (SOD) to form H_2_O_2_, thus expecting to serve as an effective support for CDT 120-122. Packaging strategies for the co-delivery of CDT agents with chemotherapeutic drugs inevitably suffer from insufficient drug loading, premature leakage, and limited reproducibility, leading to catalytic effects of H_2_O_2_ in cells and inefficiency 40, 123. In addition, chemotherapy agents used in clinical settings often lack selectivity and sensitivity towards tumor cells, leading to toxicity in normal cells, multidrug resistance (MDR), suboptimal pharmacokinetic profiles, and other limitations that hinder treatment efficacy, consequently resulting in severe adverse effects. Therefore, it is expected to construct ferrocene-based nanoplatforms incorporating chemotherapeutic drugs to decrease the adverse effects of chemotherapy on healthy tissues and enhance the CDT efficiency on tumor tissues.

**Figure 4:**
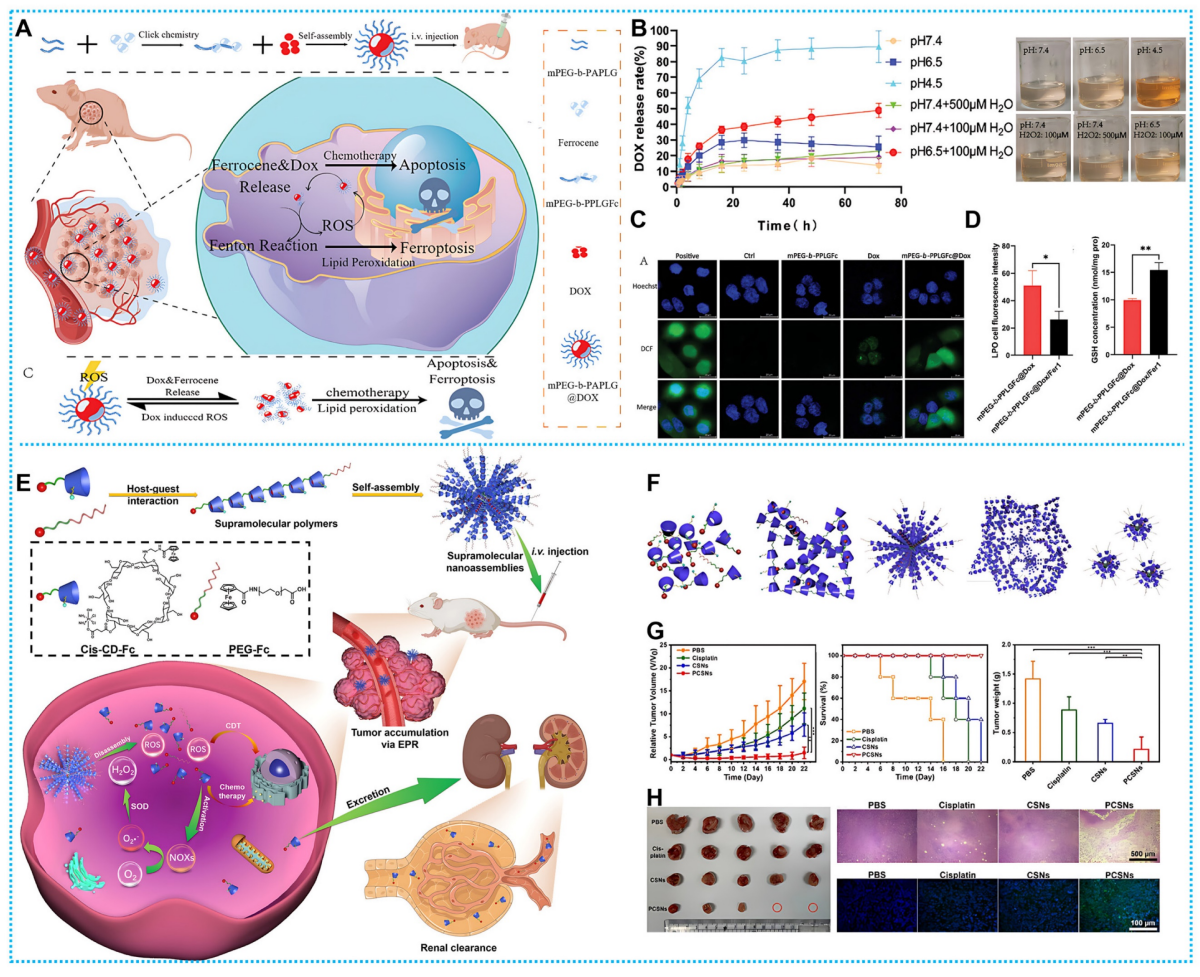
(A) Synthesis process and therapeutic mechanisms of mPEG-b-PPLGFc@Dox. (B) The dox release rate of mPEG-b-PPLGFc@Dox and variations in coloring of the liquids after 72 hours. (C) The degree of ROS in various treatments (D) LPO and GSH quantities in various treatment conditions. Reproduced with permission from ref 99. Copyright 2023, Wiley-VCH. (E) Working principle and preparation of cis-CD-Fc. (F) Representative TEM images of cis-CD-Fc. (G) The tumor volume, survival, and final tumor weight curves. (H) photographs of the dissected tumors and H&E, TUNEL staining image. Reproduced with permission from ref 100. Copyright 2021, Wiley-VCH.

Based on the above design concepts, Lin and colleagues designed mPEG-b-PPLGFc@Dox, an amphiphilic polymeric nanoparticle (**Figures 4A**). After the mPEG-b-PPLGFc@Dox reached tumor cells, endogenous H_2_O_2_ degraded it, releasing Dox and upsetting the redox equilibrium (**Figures 4B**). Fe (II) produced from ferrocene initiated the Fenton reaction, which resulted in ·OH and ferroptosis (**Figures 4C-D**). The absence of stimulus reactivity in normal tissue decreased Dox's organ toxicity 99. In addition to DOX, platinum drugs in combination with ferrocene have also reported very successful paradigms. For instance, Wang's team developed an H_2_O_2_-responsive nanomedicine cis-CD-Fc. Self-assembled cis-CD-Fc monomers with Fenton catalyst Fc as the guest and CD as the host (**Figures 4E-F**). The cis-CD-Fc obtained exhibits a substantial drug loading capacity and allows for the adjustable ratio of platinum (IV) complexes to Fc. Therefore, the medication is released by means of a sequential process of generating and dissolving reactive oxygen species (ROS) (**Figures 4G-H**) 100.

There are also cases of synergistic application of classical chemotherapeutic agents, such as Liang's team developed a small molecule prodrug (Nut@FSSG) consisting of Fc and Gemcitabine (GEM). In TME, FSSG can trigger iron death to generate LPO, leading to apoptosis. Overexpression of GSH triggers the cleavage of disulfide bonds in FSSG, which induces the decomposition of nano prodrugs, activates GEM, and releases nutlin-3a. The released nutlin-3a not only activates the p53 signaling pathway to enhance apoptosis of GEM but also inhibits cystine uptake and promotes iron death of Fc. FSSG achieves co-delivery of the cystine uptake inhibitor, Nutlin-3a, with the chemotherapeutic drug, GEM, and the iron-death drug, Fc, in the nano-remedy, which decreases chemoresistance in GEM and the pancreatic ductal adenocarcinoma (PDAC) treatment yielded good anti-tumor efficiency 101.

### 3.3. ECDT in combination with thermotherapeutic

Thermotherapy is a non-invasive cancer treatment that is primarily based on external energy sources such as photos or magnets that cause an elevation of the internal temperature of the cancerous tissue. When the local temperature of the tumor increased to over 42 °C, malignant cells could be ablated sufficiently 124. Thermotherapeutic also boosts the catalytic efficiency of ferrocene-based Fenton reaction. The therapeutic concept of combining ferrocene-based ECDT with Thermotherapeutic is proposed by constructing ferrocene-based nanomaterials. Notably, PTT causes an area-specific temperature rise that significantly speeds up the circulation inside the tumor., thereby increasing the oxygen level in the tumor region and alleviating hypoxic conditions to facilitate CDT owing to the Fenton reaction strongly depends on the involvement of oxygen for the generation of H_2_O_2_ 125.

**Figure 5:**
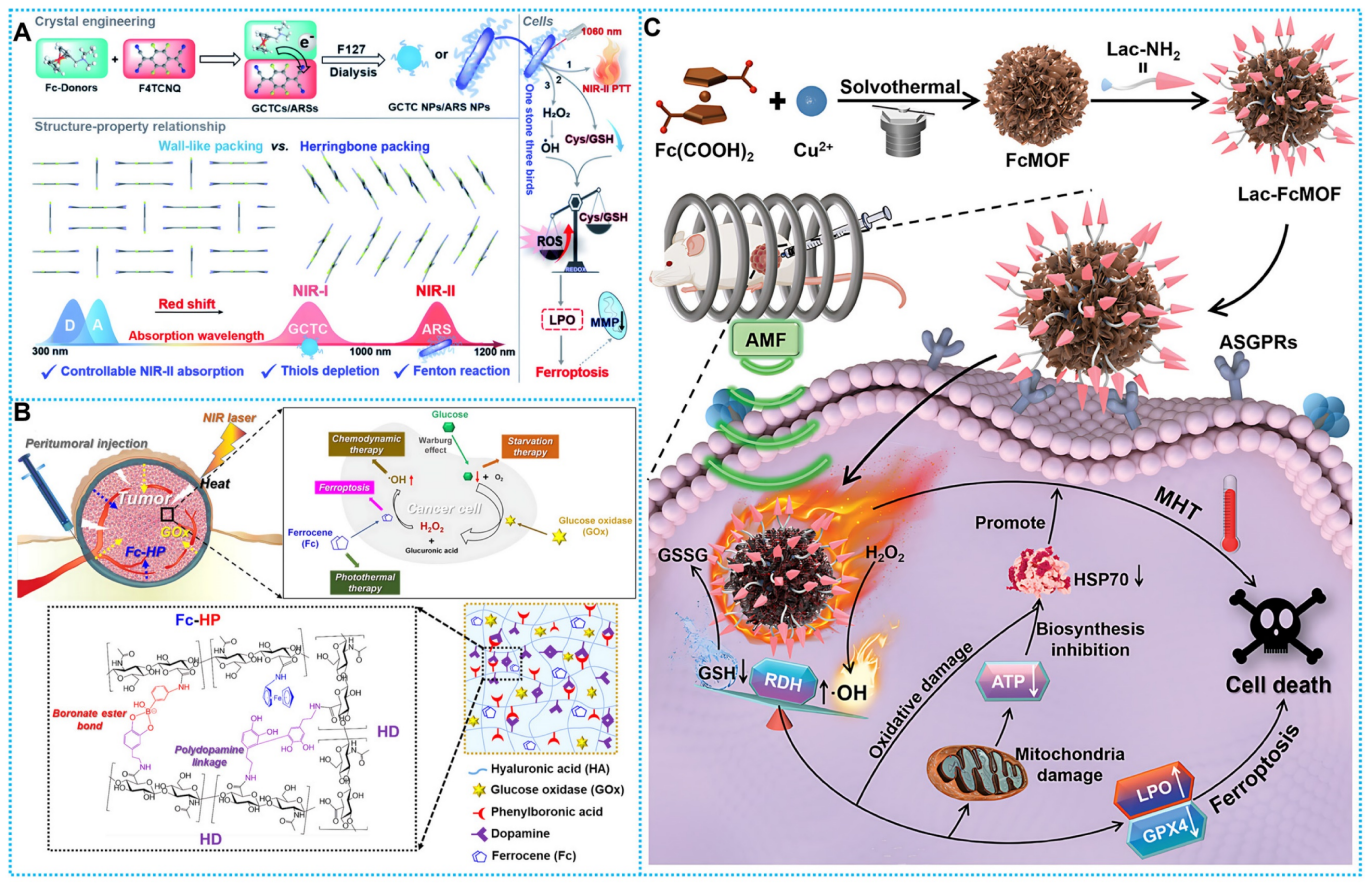
(A) Enhanced the Fc-based complex and its anti-tumor mechanism through schematic representation. Reproduced with permission from ref 102. Copyright 2022, RSC. (B) Schematic diagram of therapeutic mechanisms of Fc-HP/HD/GOx. Reproduced with permission from ref 103. Copyright 2022, Elsevier. (C) Schematic description of the fabrication and anticancer mechanism of Lac-FcMOF. Reproduced with permission from ref 104. Copyright 2024, Wily.

Based on this concept, Dong's team fabricated a variety of complexes utilizing F4TCNQ, employing ferrocene and its derivatives as electron donors. These ferrocenyl charge-transfer complexes (CTCs) exhibit photophysical properties that are contingent upon their stacking behavior. Notably, anion-radical salts (ARS) NPs like FcN-F4, FcNEt-F4, and FcNOH-F4 NPs exhibit a linear dimer configuration with close π-π stacking distances. This arrangement confers upon the nanoparticles a remarkable NIR-II absorptivity and exceptional photothermal effects upon exposure to 1060 nm laser irradiation. Furthermore, ARS NPs showcase characteristics conducive to the biothiol/H_2_O_2_ cascade reaction. This prompts GSH depletion, accumulation of ROS/LPO, and facilitates ROS-mediated cellular iron death. These discoveries stem from the crystal engineering of CTCs and offer valuable insights into the mechanisms governing optimal NIR-II uptake (**Figure 5A**) 102.

However, conventional ECDT/PTT needs to be performed multiple times. Thus, reducing the frequency of administration can improve patient compliance. Based on this concept, Lee *et al.* developed crosslinked injectable hydrogels based on opioid amine polymerization Fc-HP/HD/Gox. In anticancer applications, Fc and GOx can be sustainably released from the crosslinked hydrogel reactor system, and it contributes to effective tumor growth inhibition. Hydrogels containing Fc-HP/HD/GOx, when absorbed by a tumor following exposure to NIR laser, might potentially enhance the efficacy of breast cancer therapy by enabling a more precise control method, including ST/CDT/PTT/ferroptosis. (**Figure 5B**) 103 Further, Magnetic thermotherapeutic methods have proven to be highly effective in delivering heat to targeted areas. Pei's research team has pioneered a groundbreaking advancement by developing the first-of-its-kind lactose derivative-modified paramagnetic FcMOF, termed Lac-FcMOF. Lac-FcMOF exhibits dual functionality, possessing both magneto-thermal properties and exceptional thermal stability. Lac-FcMOF demonstrates remarkable efficacy in targeting HepG2 cells and modulating bi-directionally regulated RDH, a crucial pathway in cellular function. In addition, the porous nature of FcMOF provides enhanced drug-carrying properties in comparison to other ferrite materials. This presents new opportunities for integrating magnetothermal therapy with other therapeutic modalities, therefore providing a viable strategy for synergistic tumor treatment (**Figure 5C**) 104.

### 3.4. ECDT in combination with PDT

Photodynamic therapy (PDT) depends on laser irradiation at a specific wavelength to excite the photosensitizer delivered into the tissue. The photosensitizer in the excited state transfers the energy to the surrounding oxygen, then generates highly reactive ROS to induce oxidative reactions with neighboring biomolecules to cause cellular damage and even death. Unlike oxygen-dependent PDT, ferrocene-based CDT operates irrespective of local oxygen levels. Moreover, the initiation of ferrocene-based CDT relies on endogenous chemical stimulation instead of external energy input, thereby circumventing rapid energy dissipation 126. Furthermore, the key challenge of PDT combined with ferrocene-based CDT therapy is the TME's low oxygen level and restricted supply of hydrogen peroxide. Consequently, this field has emerged as a focal point for scientists to investigate.

**Figure 6:**
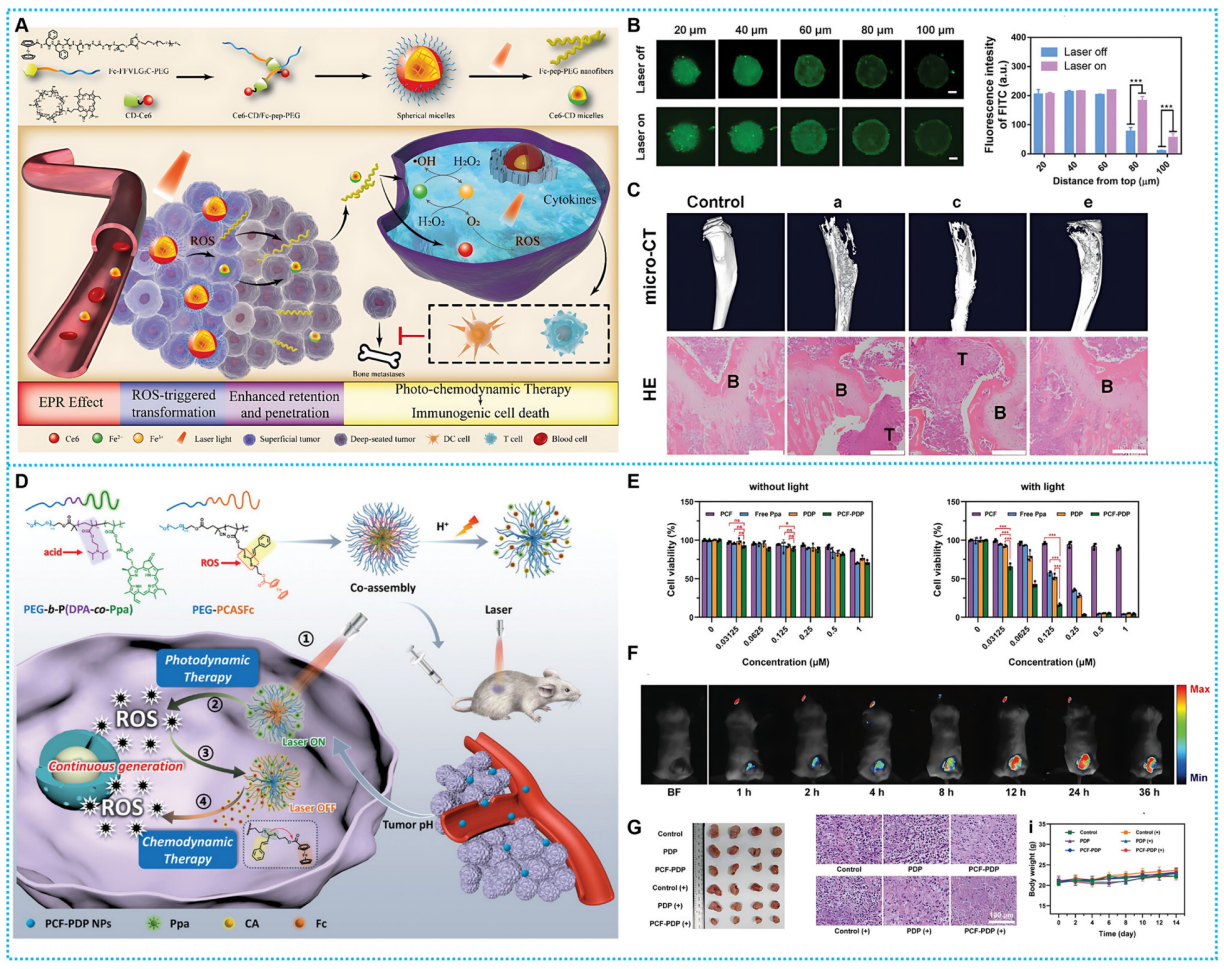
(A) Synthesis process and therapeutic mechanisms of Ce6-CD / Fc-pep-PEG. (B) CLSM of 4T1 MSCs incubated with Ce6-CD/Fc-pep-PEG-FITC (4 h) and fluorescence semi-quantification. (C) Micro-CT images and H&E staining images of the metastatic tumor-bearing tibias. Reproduced with permission from ref 105. Copyright 2021, Wiley-VCH. (D) Synthesis route and workflow of PCF-PDP NPs. (E) Cell viability at various treatments. (F) FL of mice injected with PCF-PDP NPs. (G) Evaluation of treatment effects of PCF-PDP NPs Reproduced with permission from ref 106. Copyright 2023, Wiley-VCH.

Based on this concept, Qin *et al.* constructed a self-delivery supramolecular nanoplatform with shape-shifting ability (Ce6-CD/Fc-pep-PEG). Ce6 is a good photosensitizer for mediating PDT, converting oxygen to H_2_O_2_ in response to light. The material undergoes a morphological transformation stimulated by ROS, and after passive accumulation mediated by the enhanced permeability and retention (EPR) effect, Fc is oxidized by a large amount of ROS within the tumor tissue to Fc^+^. Fc^+^-pep-PEG dissociates from the hydrophobic cavities of Ce6-CD and reassembles into nanofibers, which increase the accumulation in the tumor. At the same time, Ce6-CD fragments maintain the morphology of spherical micelles with smaller sizes and penetrate deeper into the tumor **(Figure 6A-B)**. Through this strategy, Ce6 goes deep into the tumor for PDT, and more H_2_O_2_ is generated. In response, the conserved Fc may stimulate the Fenton reaction chain reaction, which produces •OH and O_2_, boosting PDT's effectiveness and establishing an adverse reinforcement cycle. Meanwhile, combining CDT with PDT also induces immunogenic cell death (ICD), induces the maturation of DC cells and the activation of T cells, and thus inhibits the expansion of initial tumors and bone metastases (**Figure 6C**) 105.

The poor selection might be the cause of CDT's systemic adverse effects. In view of this, developing a therapeutic strategy that can consistently and successfully create ROS while retaining an excellent degree of specificity remains challenging. Based on this line of thought, Zhang and coworkers reported a ROS nano-amplifier (PCF-PDP NPs) that can be designed to induce photoactivated persistent ROS generation via the PDT-CDT chain reaction to enhance cancer therapy. Under light irradiation, PCF-PDP NPs generate a significant quantity of ROS, which may be utilized to damage malignant cells and disrupt the particles' thioacetal junction, releasing the functional molecule cinnamaldehyde (CA) and the organocatalyst Fc simultaneously (**Figures 6D**). Hence increasing the rate of •OH production in the -Fc-catalyzed Fenton reaction and the therapeutic efficacy of CDT without the presence of light. It was possible to get around the limitations of the ROS-based treatment strategy and avert harm to tissue from overboard laser irradiation by utilizing a positive feedback loop of light-triggered production of ROS, ROS-responsive prodrug stimulation, and Fenton-mediated ROS cyclic regeneration amplifiers (**Figures 6E-G**) 106.

### 3.5. ECDT based on the TME-activated strategies

Developing tumor microenvironment (TME)-activated therapeutic agents continues to pose a formidable challenge. The agents in question possess both diagnostic and therapeutic properties, with the ability to "trigger" inside the tumor and "inhibit" within normal tissue. At present, the prevailing approach typically depends on a multi-component encapsulation nanoplatform. Nonetheless, this strategy could encounter potential instability within the intricate *in vivo* environment. Therefore, investigating single-component TME-activated phototheranostic compounds could provide a way around this problem. Concurrently, biosafety stands as a significant hurdle in the progression of cancer phototherapy 127, 128. At present, the majority of TME-activated agents comprise inorganic metals and organic polymers 129. However, the potential toxicity arising from the release of metal ions poses a significant barrier to their clinical translation. Therefore, the development of a molecular phototheranostic platform with robust stability and biocompatibility emerges as a logical strategy for advancing TME-activated phototheranostic agents in multimodal cascade therapy. Recent years have also yielded very prospective research discoveries on ferrocene-based activated nanopreparations.

**Figure 7:**
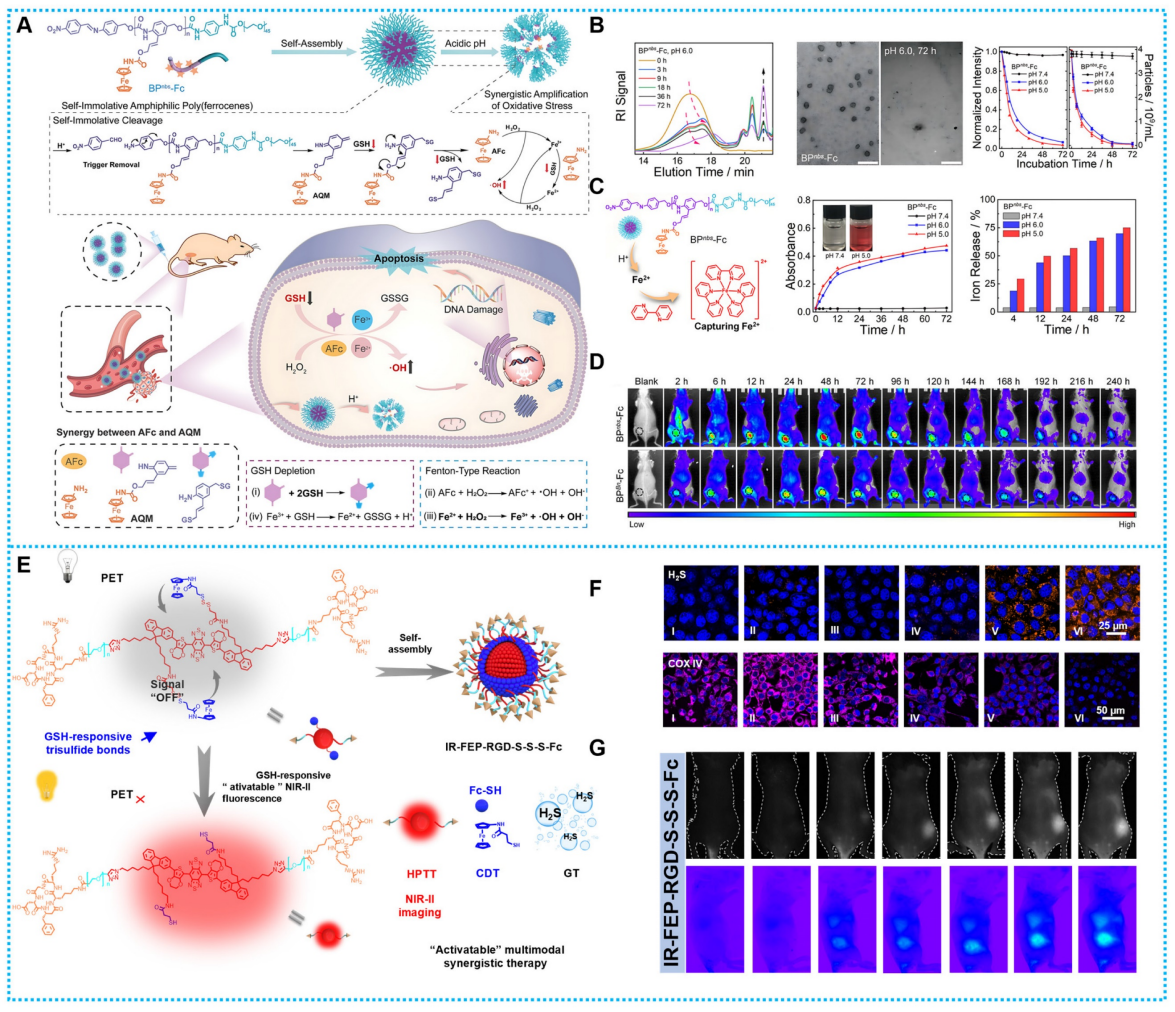
(A) Synthesis process and therapeutic mechanisms of BP*^nbs^*-Fc. (B) GPC date for the micellar dispersion, TEM images of the dispersion of BP*^nbs^*-Fc, and Time-dependent changes of BP*^nbs^*-Fc with different treatments. (C) Reaction scheme of iron ions produced from BP*^nbs^*-Fc, time-dependent development of absorbance intensity of BP*^nbs^*-Fc at different pH values, and BPnbs-Fc iron ions *in vitro* under various treatments. (D) NIR FL image after intravenous injection of BP*^nbs^*-Fc and BP*^Bn^*-Fc. Reproduced with permission 69, copyright 2023, Wiley-VCH. (E) Activation mechanism of IR-FEP-RGD-S-S-S-Fc. (F) CLSM images of Intracellular H_2_S and COX IV. (G) NIR-II fluorescence images of IR-FEP-RGD-S-S-S-Fc. Reproduced with permission 78, copyright 2023, Wiley-VCH.

Based on these challenges, Xu and colleagues recently published research on self-immolating amphiphilic poly(ferrocene) that, in acidic settings, may self-promote ROS bursts by depolymerizing and releasing highly payloaded derivatives of azaquinone methide (AQM) and aminoferrocene (AFc) fractions. Specifically, AQM-derived products can quickly and effectively react with tumor cells' cytoplasmic GSH to lower internal GSH stages, greatly increasing the susceptibility of tumor cells to oxidative stress (**Figures 7A-B**). Additionally, when AFc used its catalytic abilities, it interacted to create free iron ions, enabling the intracellular recycling of iron ions, which partially resolved the difficult issue of Fc-based nanoparticle inefficiency. Concurrently, endogenous hydrogen peroxide (H_2_O_2_) in tumor cells was transformed into highly reactive hydroxyl radicals (•OH) by AFc and Fe^2+^, which functioned as efficient Fenton reagents, damaging intracellular materials *in situ*. A logical combination between GSH depletion and •OH explosion in tumor CDT is made possible by the powerful self-incineration degradation of amphiphilic poly(ferrocene) (**Figures 7C-D**) 69.

Therefore, creating a molecular phototheranostic platform that is both stable and biocompatible is a sensible idea for creating a multimodal cascade treatment led by a TEM-activated strategy. Our research team has created IR-FEP-RGD-S-S-S-Fc based on this idea for improved gas-chemo-photothermal synergistic treatment that is GSH-activatable and guided by NIR-II imaging. Through a variety of chemical synthesis designs and spectroscopic studies, this study performed the first investigation of the link between PET efficiency and molecular spacing. The IR-FEP-RGD-S-S-S-Fc produced tumor site cascade-specific lighting by blocking the PET process by GSH shearing the trisulfide bond and accomplishing active tumor targeting via cRGDfk peptide (**Figures 7E**). Adenosine triphosphate (ATP)-dependent heat shock proteins (HSPs) were shown to be less expressed when H_2_S was released (**Figures 7F-G**) 78.

### 3.6 ECDT in combination with gene therapy

Gene therapy, such as antisense therapy, is a promising treatment for cancer. Tumor-targeted gene therapy is an emerging and promising approach for the efficient treatment of cancer. Utilizing vectors in gene therapy to introduce nucleic acids into cells and modify gene expression has the potential to prevent or reverse the aggressive growth of tumors. Global clinical studies for gene therapy are on the rise. Despite tremendous advancements in tumor-selective delivery systems over the last two decades, the generation of therapeutic vectors based on promoters that are exclusively expressed in cancer cells remains a challenging task. Consequently, several approaches have been developed that use certain gene enhancers, promoters, and 5′-untranslated regions, which are responsive to transcription factors that specifically target tumors. These tactics aim to increase the expression of tumor suppressor genes or decrease the expression of cancer-related genes 130. Refinements in genetics and molecular biology have illuminated the intricate relationship between cancer development and a myriad of genetic anomalies. Cancer gene therapy involves the delivery of targeted nucleic acids to tumor cells to correct or alter genetic conditions 131. In the past few decades, there has been a proliferation of gene therapies for various types of cancers 132, 133. It is noteworthy that gene therapy is a promising cancer treatment with excellent effectiveness with few adverse effects 134. Therefore, the combination of gene therapy and Ferrocene-based ECDT can achieve highly effective tumor treatment with few side effects.

**Figure 8:**
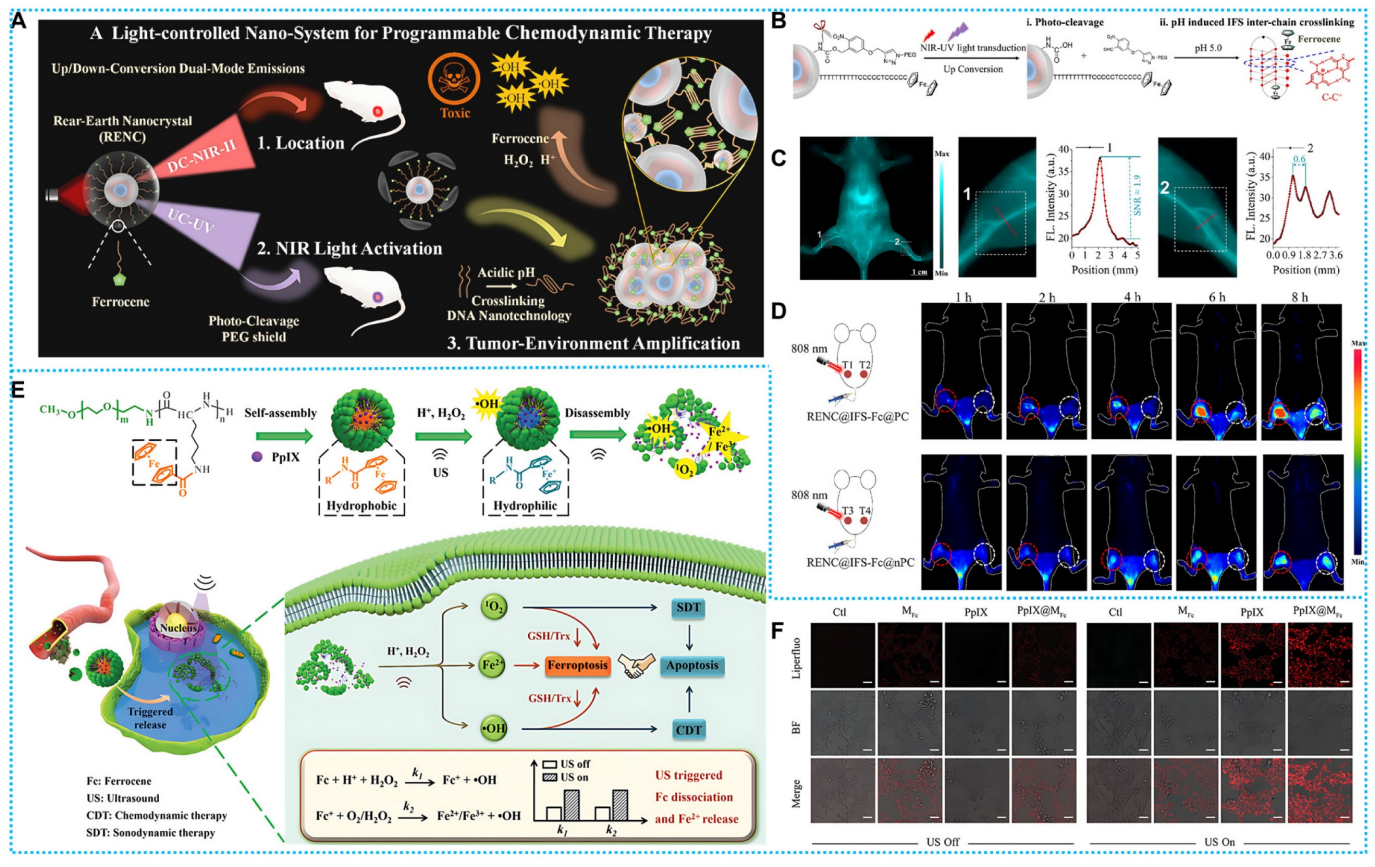
(A) Synthesis process and therapeutic mechanisms of RENC@IFS-Fc@PC. (B) Flow chart of PEG cleavage and pH response of IFS-Fc. (C) NIR-II FL imaging and peripheral tissues. (D) NIR-II FL imaging of RENC@IFS-Fc@PC and RENC@IFS-Fc@nPC. Reproduced with permission 77, copyright 2023, ACS. (E) Synthesis route and workflow of PpIX@*M*_Fc_ (F) LPO under different experimental conditions. Reproduced with permission 63, copyright 2022, Wiley-VCH.

For example, Wei *et al.* modified Fc on a DNA strand to act as a linker arm, effectively coupling Fc to the surface of rare earth nanocrystals (RENC@IFS-Fc@PC). The surface-formed photocleavable PEG network protects the IFS from degradation and “locks in” the toxicity of iron ions in the circulation (**Figure 8A**). Upconverted UV light stimulates the catalytic activity of Fe^2+^ by stripping off the PEG network. Down-converted NIR-II fluorescence can localize tumors, allowing for precise treatment of tumors (**Figure 8B-C**). This method enhances the accumulation of Fe^2+^ in tumors by altering the Fe delivery mode, which has been shown to result in a 4.5-fold increase in tumor accumulation by the nanosystem (**Figure 8D**) 77. Although this design offers a unique and effective treatment concept, the discussion on the biosafety of the gene therapy issue has been limited. In general, it is crucial to do future safety studies.

### 3.7 ECDT in combination with SDT

Chemodynamic therapy (CDT) is a novel treatment approach that is driven by the natural characteristics of tumor microenvironments, such as acidity and hydrogen peroxide (H_2_O_2_), to produce a harmful hydroxyl radical (•OH) through a Fenton or Fenton-like reaction 20-23. Extensive study has shown that, apart from Fe^2+^, other metal ions such as Mn^2+^, Cu^2+^, and Ti^3+^ may also function as Fenton-type agents and exhibit exceptional catalytic capabilities 24. SDT is a novel treatment similar to PDT that eliminates cancer cells by generating large amounts of ROS under US irradiation of a sonic sensitizer. Since ultrasound has a greater ability to penetrate biological tissues, SDT is effective in treating deep-seated tumors. However, O_2_-dependent SDT may be completely ineffective due to hypoxia and increased drug resistance in tumor tissues. Both CDT and SDT are tumor therapeutic paradigms that rely on ROS. External sonication can enhance the production of •OH by directly affecting unstable molecules or through indirect thermogenesis, thereby enhancing CDT. Therefore, combining CDT with SDT is a highly recommended cancer synergistic strategy. The rapid advancement of nanotechnology may effectively establish a connection between CDT (Cancer Diagnosis and Treatment) and SDT (Sonodynamic Therapy) 25.

Based on the above concept, Li and coworkers designed a novel “jumping frog” polymer micelle (PpIX@*M*_Fc_). After sonication, both ^1^O_2_ and •OH will be produced via a combination of SDT and CDT is used to activate apoptosis. Meanwhile, ROS-induced GSH depletion can trigger iron death, which can be augmented by the conversion of Fc to Fe^2+^ after sonication in the presence of H_2_O_2_ (**Figures 8E-F**). In addition, the triggered dissociation of Fc enables the triggered release of the acoustic sensitizer (PpIX), which also facilitates the short half-life and the limited diffusion distance of therapeutic ROS 63.

### 3.8 ECDT in combination with IT

Cancer immunotherapy (IT) has shown considerable promise in the treatment of solid tumors by stimulating the innate immune system to limit tumor growth 135, 136. In this case, increased pro-tumoral M2 macrophage polarization and anti-tumoral M1 phenotype, together with increased hydrogen peroxide from polarized M1 macrophages, produce immunogenic TME, whereas Fenton/Fenton-like reactions are enhanced 137, 138. Tumor cells undergo ferroptosis when exposed to high concentrations of •OH produced by NIR-II irradiation. Simultaneously, exposure to tumor antigens causes cell death, which raises TME's immunogenicity. This results in a two-way loop of immunological and CDT, improving the persistence that is lacking in traditional CDT therapy.

**Figure 9:**
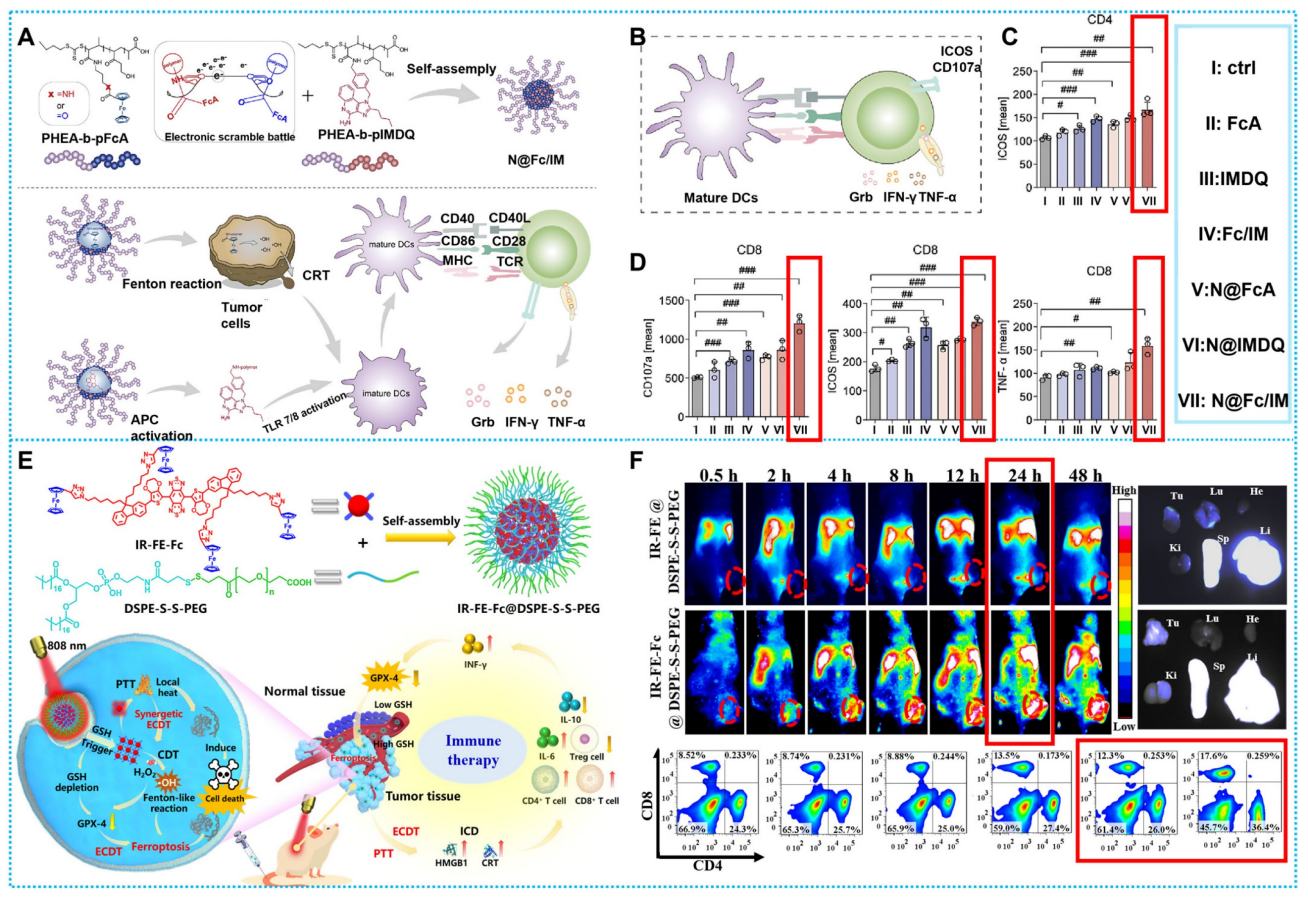
(A) Synthesis of pHEA-b-pFcAam and pHEA-b-pIMDQ and CDT destroys tumor cells and further induces ICD and enhances immune response. (B-D) Schematic representation and timing of important proteins or substances expressed or secreted by stimulated T cells. Reproduced with permission 107, copyright 2023, Elsevier. (E) Synthesis and therapeutic modalities of IR-FE-Fc@DSPE-S-S-PEG. (F) Tumor *in vivo* imaging and immune changes. Reproduced with permission 108, copyright 2023, Elsevier.

According to current studies, CDT may elicit an immunological response by means of immunogenic cell death (ICD), a particular cancer-killing mechanism 139. Exposure to calreticulin (CRT), referred to as the “eat-me” signal, induces dendritic cell (DC) maturation, which stimulates T lymphocyte activation 32. Nevertheless, ICD alone is unable to sufficiently stimulate immunity that kills tumors, and new research indicates that immunological adjuvant combinations may be helpful 140. Based on this concept, Zhang's Team developed a polymer platform (N@Fc/IM). By breaking down H_2_O_2_ to create •OH, Fc in N@Fc/IM promoted the Fenton reaction, which upset the critical redox equilibrium and caused the ICD of tumor cells exposed to CRT. Subsequent to IMDQ-induced activation of TLR7/8, the matured dendritic cells (DCs) enhanced T-lymphocyte infiltration, thereby enhancing a robust antitumor immune response (**Figures 9A**). Using four female BALB/c mouse models carrying T1, N@Fc / IM exhibited potent therapeutic effects and triggered a strong immunological response within the tumor microenvironment (TME) *in vivo* (**Figures 9B-D**) 107. Nevertheless, the correlation between the immune response and Fenton's therapy, as well as other diagnostic and therapeutic processes, has not been extensively researched. Therefore, there is a need to delve further into the specific mechanisms that are directly linked to it.

Impressively, Wu and colleagues used a straightforward and practical rational design method to build IR-FE-Fc@DSPE-S-S-PEG 108. By producing photothermal effects and converting less endogenous reactive hydrogen peroxide (H_2_O_2_) into •OH, the nanoplatform may kill tumor cells and cause tumor iron death by depleting GSH. Notably, it was shown that exposure to laser radiation had markedly higher levels of CD4^+^ and CD8^+^, indicating that the mice's autoimmunity was, in fact, enhanced after the combined therapy (**Figures 9E-F**) 108.

Liu's team has introduced a groundbreaking cancer nanobomb, termed ig, which comprises a (Pt (IV)) prodrug (Artoxplatin) and an oxidatively cleavable poly igniter embedded with Fc units. The nanobomb^ig^ liberates Oxaliplatin (Oxa), artesunate. (ART), and Fc, thereby initiating a cascade leading to the production of highly cytotoxic ROS through the catalytic reaction of ART and Fc. This amplifies cytotoxicity and augments ICD effects, culminating in the induction of robust anti-tumor immunity. Furthermore, metabolomics analysis reveals that the nanobomb^ig^ dramatically alters glutamine-related metabolic pathways, consequently reducing the levels of glutathione (GSH) within cancer cells. These findings underscore a promising strategy to bolster cancer chemoimmunotherapy when combined with immune checkpoint blockade, thus offering significant potential for clinical implementation and translational advancement 109.

### 3.9 ECDT in combination with GT

Gas therapy (GT) is an innovative treatment approach such as treatment with Nitric oxide (NO), hydrogen (H_2_), 141 hydrogen sulfide (H_2_S),^21^ and carbon monoxide (CO) 23. Due to its inherent connection with different metabolic pathways, the accessible gas, when present at optimal concentrations, exerts an anticancer impact by suppressing the production of HSPs and reversing the Warburg effect in cancer cells without negatively affecting normal cells. Thus, the integration of ferrocene-based CDT with GT holds promise in realizing a synergistic HPTT approach with heightened efficacy against malignant tumors. In addition, certain reducing agents, such as H_2_S, can expedite the conversion of Fe^3+^/Fe^2+^, thereby augmenting •OH generation and enhancing the therapeutic potential of CDT.

**Figure 10:**
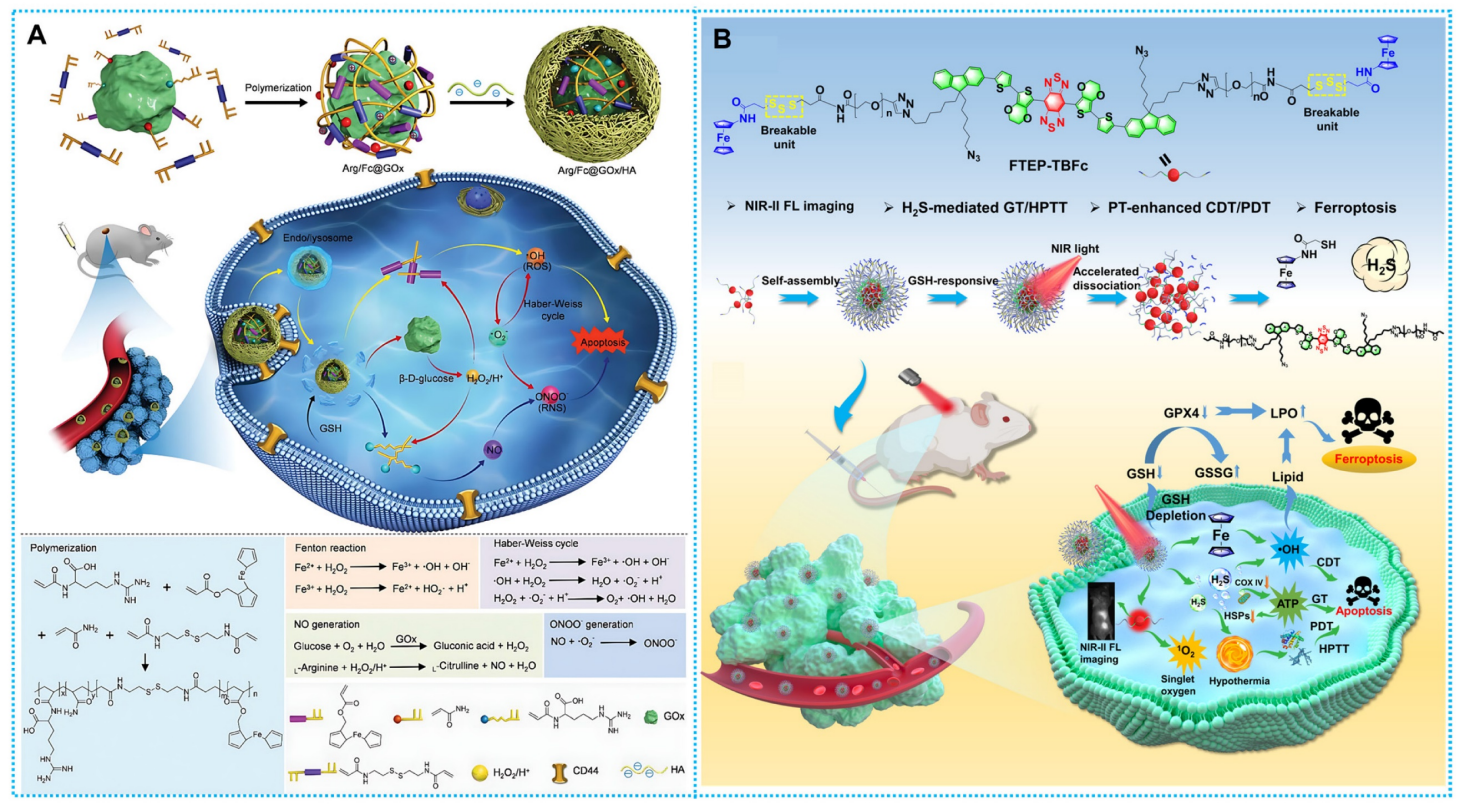
(A) Synthesis and therapeutic mechanisms of Arg/Fc@GOx/HA. Reproduced with permission 111. Copyright 2023, Wiley-VCH. (B) Synthesis and therapeutic mechanisms of FTEB-TBFc. Reproduced with permission 110, copyright 2023, ACS.

Nitric oxide (NO) radicals are the main kind of reactive nitrogen species (RNS) that damage mitochondria and DNA and may immediately trigger apoptosis 142, 143 and subsequently increase the effectiveness of PDT and SDT via quickening the metabolism of intracellular glutathione (GSH) and widening blood arteries to provide oxygen 144, 145. More importantly, NO has further reactions with ROS (e.g., •O_2_^-^) to produce peroxynitrite anions (ONOO-), 146 another RNS that is more hazardous than the majority of ROS since it may cause direct oxidation and nitration processes mediated by free radicals 147, 148. Inspired by the natural mechanism by which inflammatory cells generate a storm of free radicals through activated and tunable biocatalysis, Liu *et al.* developed a biomimetic nano-enzyme capable of generating reactive oxygen and nitrogen species (RONS) *in situ* for cancer therapy. The nano-enzyme gets separated by internal glutathione, releasing GOx, which breaks down glucose to generate gluconic acid and H_2_O_2_. Under this acidifying condition, H_2_O_2_ efficiently oxidizes side-chain arginine residues to generate nitric oxide, which is converted to Highly hazardous hydroxyl radicals and superoxide anion. This process mainly produces peroxynitrite anion (ONOO-). This reactive nitrogen (RNS) is more toxic than most ROS. Enhanced tumor-killing effect (**Figure 10A**) 111.

Furthermore, the activatable nanomaterials would be a dependable and effective tumor treatment with excellent effectiveness, powerful targeting, flexible controllability, and few side effects. The logical design of FTEP-TBFc NPs was shown by Wu *et al.* based on this concept. This molecularly engineered photothermal nanoplatform produces hydrogen sulfide (H_2_S) gas in response to glutathione (GSH) and transports ferrocene molecules into the TME. This strategy improves photodynamic treatment (PDT) and chemodynamic therapy (CDT) at a biosafe laser power of 0.33 W/cm^2^** (Figure 10B)** 110.

## 4. Summary, Prospects, and Challenges

This article summarizes the latest research progress of ferrocene-based nanomedicines for synergistically enhanced chemodynamic therapy. Ferrocene-based chemodynamic therapy has several distinct advantages of excellent stability, high efficiency, flexible functionalization, and low toxicity, exhibiting brilliant prospects for oncological applications. For the first time, this paper focuses on the different types of ferrocene-containing nanomedicines and describes the relationship between the functionality and various states of ferrocene that exist in nanomedicine, followed by recent progress in cancer-enhanced chemodynamic therapy applications with other treatment patterns. Additionally, the recently reported Ferrocene-based ECDT nanoplatform is highlighted, shedding light on the molecular mechanisms behind reinforcement strategies to promote the development of ferrocene-based ECDT anticancer methods. But before they may be used in clinical settings, some unresolved concerns need to be addressed, which are listed as follows.

(1) The therapeutic mechanisms of ferrocene-based CDT in complicated TME should be thoroughly elucidated. Currently, most of the research on the mechanism and properties of the Fenton reaction has been performed in simulated humoral environments, which may differ from intricate physiological circumstances. It is foreseeable that abundant enzymes, proteins, and bioactive molecules are likely to inhibit the Fenton reaction under pathological conditions. In addition, due to tumor heterogeneity, the therapeutic approach should be individualized for different tumors. Therefore, there is an urgent need for an in-depth study of the response mechanism of ferrocene-based CDT under specific pathological conditions, which requires targeted cellular or *in vivo* models for effective validation. Meanwhile, the creation of an online immediate form ROS auditing system to direct the logical design of upcoming effective Fenton reagents.

(2) The controllable manufacture of ferrocene-based nanomedicines with active ingredient encysted and the species of ferrocene-based CDT drugs and the regimen for dosage are tailored to different patients for meeting the next phase of clinical medicine 149. So far, the efficiency of ferrocene-based CDT is closely related to the TME and biochemical reaction, which changes across tumor varieties and phases, as well as individuals 150, 151. The good news is developments in big data, artificial intelligence (AI), and tumor whole-genome sequencing technologies may enable us to build a multivariate database that crosses TME, spatiotemporal distribution of nanomedicines, and ferrocene CDT results. Moving forward, data-driven nano bioinformatics has great prospects for guiding the design of customized ferrocene CDT nanopharmaceuticals and achieving precise ferrocene CDT.

(3) Leakage of ferrocene-based nanomedicines with releasing ferrocene into the bloodstream would cause toxicity and inefficient treatment. In general, Transition ferrocene-encapsulated nanoplatforms have been suffering from ferrocene leakage problems. Consequently, Monitoring blood Fe concentrations and long-term *in vivo* toxicity are essential. The majority of the existing ferrocene nanoplatforms are molecularly encapsulated. Recent investigations suggest an evolution towards completely manufactured, chemically coupled, single-molecule medicines, which provide the benefit of guaranteeing stability inside a living organism. Simultaneously, it is necessary to conduct realistic and thorough toxicological assessments, as well as clinical and preclinical investigations, in order to provide a foundation for the ongoing development of future medications.

(4) The biodegradability and biosafety of ferrocene-based agents remain to be major concerns. Despite a number of studies that have examined the safety and biocompatibility of nanocarriers in pathology and blood, however, there are currently insufficient long-term safety evaluations across a variety of experimental models. In order to effectively translate intriguing hypotheses into pre-clinical and clinical trials, it is crucial to take into account the physical compatibility, pharmacological synergy, and pharmaceutical features such as stability, scalability, and pharmacokinetics from an early stage. By using this method and implementing strong production procedures, several drug-combination nanoparticles have advanced to trials, including non-human primates and humans. Meanwhile, several labeling methods, such as isotope labeling and Positron Emission Tomography (PET), may be used to track the destiny of nanomaterials *in vivo*. For clinical translation, a thorough safety evaluation by conducting nanotoxicity investigations on delivery platforms involving larger animals and primates is required.

(5) The development of ferrocene-based nanomedicines with low toxicity, strong tumor targeting, and immune escape remains a major challenge for CDT therapy. Despite the advancement of targeted strategies, some challenges still exist. Examples include the creation of “protein crowns” during blood transport, the immune system's rejection of nanoparticles, and the stability and targeting capacity of nanomaterials *in vivo*. In the last several years, nanobionic therapeutic platforms have received widespread attention, allowing nanomedicines to self-recognize substances and avoid recognition by the immune system. Specifically, Cancer cells' membrane surface antigen homology offers opportunities for immune evasion and homologous adhesion. Replicating these functions in full using synthetic materials is difficult. To facilitate further progress, it is recommended to develop customized nanoplatforms by the loading or chemical coupling of cancer cells obtained from patient tissues utilizing biomimetic techniques. This novel design is anticipated to enhance the targeting efficacy of the treatments and circumvent detection by the immune system.

(6) Ferrocene-based activatable theranostics with the integration of diagnosis and treatment function would facilitate precise tumor detection and treatment with minimal non-specific damage. Thus obtaining a high tumor-to-normal tissue (T/N) signal ratio, sound sensitivity, and avoiding “false positives.” Furthermore, they are able to overcome undesirable toxic side effects during diagnosis and therapy due to their higher selectivity. It is thus very valuable to research the activatable Ferrocene-based ECDT diagnostic and therapeutic platforms. For instance, the work put forward by our team and Chen *et al.* on activation-based research serves as a valuable reference for investigators. Activation probes having a high signal ratio between target and non-target regions, as well as excellent sensitivity, may be developed by molecular design using PET, ICT, or FRET mechanisms.

(7) To manage the tumor as a chronic condition, the ferrocene-based ECDT should focus more on creating innovative nanomedicine that targets tumor evolution. Because tumor cells' genomes are unstable, these cancerous cells may undergo natural selection in response to pressures from the tumor's survival or outside treatments 152-154. The three main benefits of nanomedicines—exact controllability, durability, and inclusivity—should be thoroughly understood in the coping strategy. In response strategies, interventions should be developed to target tumor cells or the tumor microenvironment. Regardless of how the tumor evolves, it will build up a tumor microenvironment. Investigating the evaluation and management of the tumor microenvironment has the potential to provide precise and timely diagnosis and therapy. Moreover, with the advanced technique of whole tumor genome sequencing, a thorough examination of the patient's tumor cells may be conducted to acquire a tailored diagnosis and treatment strategy, thereby addressing the challenge at present.

In summary, although much effort has been made to enhance ferrocene CDT's activity, more work has to be done prior to it entering clinical applications. Urgent issues such as safety, efficiency, stability, and reproducibility of ferrocene CDT reagent design should be fully considered. Meanwhile, unresolved scientific issues need to be resolved through interdisciplinary collaboration among clinical medicine, chemistry, pharmacy, and biology in order to further accelerate the discovery of ferrocene CDT. Since ferrocene CDT has been demonstrated to have promise for usage in medical settings in earlier studies, we anticipate that its real clinical uses will benefit a greater number of individuals.

## Figures and Tables

**Table 1:** Summary of Nanomedicine Platform centered on Ferrocene-based ECDT.

Ferrocene-based Nanomedicines	Type of Nanomedicines	Mechanism	Advantages and Shortcomings	*In vivo*	Administration Method	Enhance Strategies	Reference
GOx@T-NPs	Ferrocene-encapsulated nanomedicines	Amplifying H_2_O_2_, decreasing pH	Three-modality therapy is novel but lacks validation of tumor recurrence or not.	HT-29 cells	Intravenous injection	ST/CDT	97
GOD/TPZ@PFc	Ferrocene-coupled nanomedicines	Amplifying H_2_O_2_, decreasing pH	Tumor acid response nanoreactors exhibit better tumor targeting ability but lack studies on related mechanisms.	H22 cells	Intravenous injection	ST/CDT	98
PFW-DOX/GOD	Ferrocene-coupled nanomedicines	Photothermal energy conversion, amplifying H_2_O_2_, decreasing pH	Self-Immolative amphiphilic poly(ferrocenes) for synergistic amplification of oxidative stress in tumor therapy.	HeLa cells	Intravenous injection	ST/CT/CDT	35
Co-Fc NMOF	Ferrocene-based Metal-organic Frameworks (MOFs)	Amplifying H_2_O_2_, decreasing pH	Unique cascade enzyme/Fenton effect that promotes kinetic chemotherapy treatment, but long-term toxicity needs to be considered.	4T1 cells	Intravenous injection	ST/CDT	91
G-b-PPLGFc@Dox	Ferrocene-coupled nanomedicines	Amplifying H_2_O_2_ and depletion of GSH	Ferrocene-based polymeric nanoparticles carrying doxorubicin for combination of chemotherapy and ferroptosis, but Lack of studies on related mechanisms.	DU145 cells	Intravenous injection	CDT/CT	99
cis-CD-Fc	Ferrocene-coupled nanomedicines	Amplifying H_2_O_2_	PCSNs exhibited excellent biocompatibility and minimized side effects, which would be more convincing if validated with organoid models.	4T1 cells	Intravenous injection	CDT/CT	100
Nut@FSSG	Ferrocene-coupled nanomedicines	Depletion of GSH	Nut@FSSG case apoptosis by the p53 activation, leading to good combination therapy with GEM in PDAC treatment	Panc02 cells	Intravenous injection	CDT/CT	101
FcNEt-F4 NPs	Ferrocene-coupled nanomedicines	Thermal acceleration and GSH depletion	Nut@FSSG leads to apoptosis by activating p53, thus forming a good combination therapy with GEM in PDAC treatment, but lacks validation of tumor recurrence or not	4T1 cells	Intravenous injection	PTT/CDT	102
Fc-HP/HD/GOx	Ferrocene-coupled nanomedicines	Photothermal energy conversion, amplifying H_2_O_2,_ and decreasing pH	Fc-HP cross-linked injectable hydrogels demonstrate shear-thinning behavior, self-healing properties, and high feasibility in clinical applications but need to advance preclinical studies	4T1 cells	Peritumoral injection	PTT/CDT/ ST	103
Lac-FcMOF	Ferrocene-encapsulated nanomedicines	Thermal acceleration and GSH depletion	The Lac-FcMOF complex demonstrates efficient targeting of HepG2 cells, and its porous structure provides excellent capacity for delivering drugs. However, it requires more extensive validation to ensure its biosafety.	HepG2 cells	Peritumoral injection	MMHT/CDT	104
Ce6-CD / Fc-pep-PEG	Ferrocene-based phototheranostic platform	Thermal acceleration	Fc-pep-PEG recombined to nanofibers, and Ce6-CD maintained the form of a spherical micelle with a smaller size, enhancing the penetration and retention, but Lack of Studies on Related Mechanisms.	4T1 cells	Intravenous injection	PTT/CDT/IT	105
PCF-PDP NPs	Ferrocene-coupled nanomedicines	GSH depletion	PCF-PDP NPs could achieve ROS cascade-amplifying through the positive feedback loop for dual-dynamic cascade cancer therapy but need to advance preclinical studies	4T1 cells	Intravenous injection	PDT/CDT	106
BP*^nbs^*-Fc	Ferrocene-coupled nanomedicines	GSH depletion	BPnbs-Fc exhibits exceptional photothermal conversion efficiency by the integration of a stiff planar donor-acceptor structure with a molecular rotor, but Lack of Studies on Related Mechanisms	4T1 cells	Intravenous injection	CDT and TEM-activated therapy	69
IR-FEP-RGD-S-S-S-Fc	Ferrocene-based phototheranostic platform	Thermal acceleration, GSH depletion, and accelerated Fenton cycling.	The therapeutic benefits of IR-FEP-RGD-S-S-S-Fc are achieved by a four-mode activation design, although molecular-level mechanistic research is absent.	4T1 cells	Intravenous injection	HPTT/CDT/GT and TEM-activated therapy	78
PpIX@*M*_Fc_	Ferrocene-coupled nanomedicines	GSH depletion	PpIX@MFc were constructed to realize the controlled production of Fe^2+^, •OH, and ^1^O_2_ in the tumor site, and non-invasive deep-penetration ultrasound. , but need to advance preclinical studies.	4T1 cells	Intravenous injection	SDT/CDT	63
RENC	Ferrocene-based phototheranostic platform	GSH depletion and accelerated Fenton cycling.	DNA-modified RENCs work as a programmable remoter, providing a practical strategy for the safe and efficient delivery of Fe^2+^, showing great significance for the clinical applications of CDT, but need to advance preclinical studies	4T1 cells	Intravenous injection	CDT and gene therapy	77
N@Fc/IM	Ferrocene-coupled nanomedicines	GSH depletion.	N@Fc/IM stimulates anti-tumor immunity by triggering a potent Fenton reaction, yet a single treatment approach still has limitations.	4T1 cells	Intravenous injection	CDT/IT	107
IR-FE-Fc@DSPE-S-S-PEG	Ferrocene-based phototheranostic platform	GSH depletion	The IR-FE-Fc@DSPE-S-S-PEG compound demonstrates innovative approaches to iron-induced cell death, but the molecular mechanisms need to be investigated.	4T1 cells	Intravenous injection	PTT/CDT/IT	108
Nanobomb^ig^	Ferrocene-coupled nanomedicines	GSH depletion	The Nanobomb^ig^ system exhibits enhancing cell toxicity in tumors, but more research is required to assess its biosafety.	CT26 cells	Intravenous injection	CDT/IT	109
FTEB-TBFc	Ferrocene-based phototheranostic platform	Thermal acceleration, GSH depletion, and accelerated Fenton cycling.	FTEB-TBFc has developed diagnostic approaches that are triggered by several modes. However, a thorough safety evaluation is still lacking.	4T1 cells	Intravenous injection	HPTT/PDT/CDT/GT and TEM-activated therapy	110
Arg/Fc@GOx/HA	Ferrocene-coupled nanomedicines	Thermal acceleration, GSH depletion, and accelerated Fenton cycling.	The Arg/Fc@GOx/HA stimulates the generation of reactive oxygen and nitrogen species (RONS) free radicals inside cancer cells, effectively suppressing the growth of tumors while little affecting healthy tissues.	4T1 cells	Intravenous injection	ST/CDT/GT	111
